# Supplementary material for: MetaRibo-Seq measures translation in microbiomes
Source: Nat Commun. 2020 Jun 29;11:3268. doi: 10.1038/s41467-020-17081-z (PMC7324362; doi:10.1038/s41467-020-17081-z)
Supplement: Supplementary file 10 — Supplementary Data 7 [file 41467_2020_17081_MOESM10_ESM.zip › File2/Confidence_High_Taxonomy/409923_out.krona.html]

Javascript must be enabled to view this page.

members
magnitude
magnitudeUnassigned
count
unassigned
taxon
rank

409923\_out

3

superkingdom
2
3

3
phylum
1224

3
class
1236

order
91347
3

3
family
543

genus
561
3

562
species
3

SRS013476\_contig\_number\_20191SRS024132\_contig\_number\_11917SRS078419\_contig\_number\_19175
